# Validation of a Cariogenic Biofilm Model to Evaluate the Effect of Fluoride on Enamel and Root Dentine Demineralization

**DOI:** 10.1371/journal.pone.0146478

**Published:** 2016-01-05

**Authors:** Constanza E. Fernández, Livia M. A. Tenuta, Jaime A. Cury

**Affiliations:** Department of Physiological Sciences, Piracicaba Dental School, University of Campinas (UNICAMP), Piracicaba, São Paulo, Brazil; University of Washington, UNITED STATES

## Abstract

Due to gingival recession both enamel and root dentine are at risk of developing caries. Both tissues are exposed to a similar environment, however there is not a validated model to evaluate the effect of fluoride on these dental substrates simultaneously. Hence, this study aimed to validate a caries model to evaluate the effect of fluoride to prevent demineralization on enamel and root-dentine. *Streptococcus mutans* UA159 biofilms were formed on saliva-coated bovine enamel and root dentine slabs (n = 12 per group) mounted in the same well of culture plates. The biofilms were exposed 8×/day to 10% sucrose and treated 2×/day with fluoridated solutions containing 0, 150, 450, or 1,350 ppm F; thus, simulating the use of low to high fluoride concentration toothpastes. The pH values of the culture medium was monitored 2×/day as a biofilm acidogenicity indicator. After 96 h, biofilms were collected for fluoride concentration analysis. The percentage of surface hardness loss (%SHL) was calculated for slabs. The fluoride uptake by the enamel and dentine was also determined. The model showed a dose-response because the biofilm and fluoride uptake increased and %SHL decreased at increasing fluoride concentrations (p < 0.05). Fluoride in the biofilm formed on dentine and fluoride uptake by dentine were higher than those for enamel. With the same fluoride concentration treatment, the percentage of reduction of demineralization was lower for dentine than for enamel. In conclusion, the model was validated in terms of a dose-response effect of fluoride on enamel and root dentine. Furthermore, the findings support the clinical data, suggesting that higher fluoride concentrations are necessary to control caries of root dentine than of enamel.

## Introduction

The decrease of caries prevalence and the increase in life expectancy [1] allow more natural teeth to remain in the oral cavity in elderly. In this context, root caries is an important problem [2, 3] and the challenge is to maintain both the coronal and root caries under control. Whenever the root is exposed to the oral cavity, enamel and cervical dentine are subjected to a similar environment: biofilm formation, dietary carbohydrates exposure, and fluoride use. Nevertheless, caries progress is faster in dentine than in enamel [4] because dentine has a higher organic matrix percentage [5], higher permeability [6], and smaller crystals with higher carbonate concentration [7], resulting in a higher critical pH for dentine than for enamel demineralization [8–10]. Therefore, under the same cariogenic challenge, dentine should be considered more susceptible to demineralization than enamel.

Fluoride is still the main strategy for non-invasive control of root caries [11]. Among all methods of fluoride delivery, toothpaste is responsible for the decline of coronal caries [12, 13] and its recommendation is based on strong evidence [14, 15]. However, the knowledge about the anti-caries effect of fluoride on dentine is scarce [6, 16], and some studies suggest that it would not be of the same magnitude as that on enamel [17, 18]. For dentine, higher fluoride concentration [19–23], higher frequency of use [24], or combination of methods of fluoride delivery [25] should be necessary to control caries; however, the evidence is not conclusive.

Models are widely used to evaluate the anti-caries potential of toothpastes; however, they should show dose-response effects because there is evidence that the effect of fluoride toothpaste on caries control is concentration dependent [14, 26]. The most used models are chemical, named pH-cycling models, which simulate the caries process. pH-cycling models have been validated to evaluate the dose-response effect of standard and low fluoride toothpaste concentrations on enamel [27]. Furthermore, pH-cycling models are chemical models that are not able to estimate the antimicrobial effect of fluoride or other substances on caries. Thus, biofilm models are more suitable to evaluate the relevance of antimicrobial effects on caries. However, there is no validated pH-cycling model or biofilm model to evaluate the effect of fluoride on dentine or on both enamel and dentine.

Biofilm models should mimic bacterial accumulation on dental surfaces and its exposure to a cariogenic challenge, thereby simulating the caries process. Among several biofilms models [28–33], many still use surrogates of dental substrates [29, 31, 32] instead the use of enamel or dentine, which our research group has used [28, 33–37]. We recently validated a *Streptococcus mutans* biofilm model to evaluate the effect of antimicrobial agents on biofilm formation and enamel demineralization [28], using chlorhexidine as the positive control. This model was successfully used to evaluate the effect of iron on enamel [37] and dietary products on either enamel or dentine demineralization [34–36]. However, this model was not validated to evaluate the dose-response effect of fluoride either in enamel or dentine or simultaneously in both. Therefore, the aim of this study was to validate a cariogenic biofilm model to evaluate the effect of fluoride on enamel and root dentine under simultaneous conditions of demineralization.

## Materials and Methods

### Experimental design

This study was approved by the local Research and Ethics Committee (protocol No.108/2011). The current biofilm model was adapted from the Ccahuana-Vasquez and Cury's model [28], which was previously validated to evaluate the antimicrobial agents. This model allows to estimate the anti-caries effect of substances on enamel and dentine demineralization inhibition upon a high cariogenic challenge, and also to analyze the formed biofilm. *S*. *mutans* (SM) biofilms were formed on bovine enamel and root dentine slabs for 96 h. Biofilms/slabs were exposed 8×/day to 10% sucrose and 2×/day to 0, 150, 450, or 1,350 ppm F. These concentrations simulate the dilution (1:3) by saliva in the oral cavity during tooth brushing with fluoride toothpaste [38]; thus, simulating the use of toothpaste from low to high fluoride concentration (0, 500, 1,100, and 5,000 ppm F). The solutions were made with NaF and purified water. The medium was changed 2×/day and aliquots were analyzed to determinate the pH, F, and Ca concentration. At the end of experiment, the concentrations of water-soluble fluoride and acid-soluble fluoride were determined in the biofilms. In the slabs, the percentage of surface hardness loss (%SHL) and fluoride uptake were assessed. The study was conducted in three independent assays (n = 12/substrate/group) and response variables were blindly analyzed.

### Enamel and root dentine slabs preparation

Slabs, 7 × 4 × 1 mm in size, were obtained from the crown and cervical roots of bovine incisors [39]. Enamel slabs were obtained from the central part of the dental crown [28]. For root dentine, a 7 mm root slice was cut using two parallel diamond disks from the cementum-enamel junction, then cut to a 4 mm size mesiodistally to obtain the slab. Both substrates were flattened externally and internally on both surfaces, and the external surface was polished using 400, 600, and 1,200 grades of Al_2_O_3_ papers and polishing cloths with 1 μm diamond paste. The initial SH of slabs was determined by three indentations using a Knoop diamond indenter spaced 100 μm apart made with 50-g load for enamel and 5-g for root dentine on the polished surface, for 5 seconds (Future-Tech FM, Kawasaki, Japan). Before performing SH measurements, dentine slabs were allowed to dry for at least 30 minutes to standardize the measurements [25]. Slabs with intra-variability of SH lower than 10% were selected. Them for a stratified randomized selection slabs with a hardness of 336.2 ± 14.5 Kg/mm^2^ (n = 48) for enamel and 36.5 ± 1.7 Kg/mm^2^ (n = 48) for root dentine were included in the experiments. Ethylene oxide was used for sterilization of the slabs [40].

### Biofilm model

**Fig 1:**
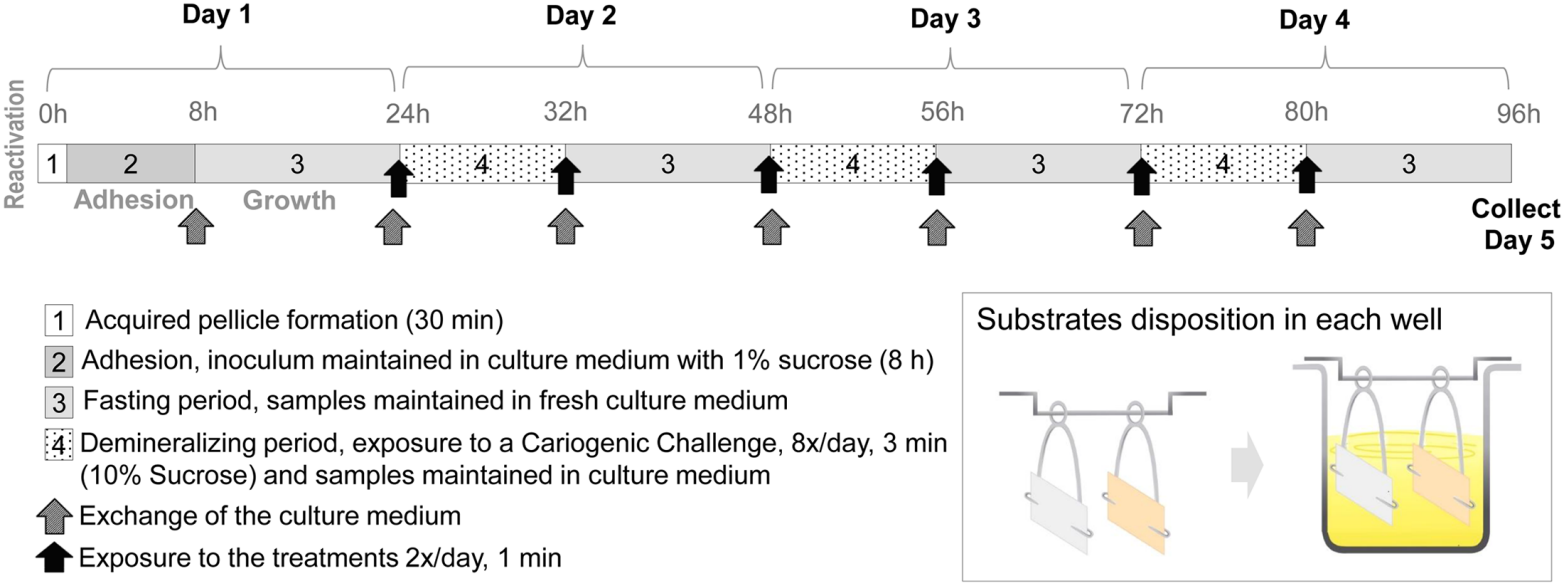
Outline of the experiment and details of enamel and dentine slabs positioning in the plate well.

*S*. *mutans* UA159 (SM UA 159) biofilms were grown for 96 h on saliva-coated slabs of enamel and root dentine with surface hardness (SH) previously determined. One slab of each dental substrate was assembled vertically using metallic holders in the same well of a 24-well culture plate (Fig 1). The slabs were immersed for 30 min in filtered human saliva [32, 33] to simulate the formation of an acquired pellicle and to facilitate bacterial adhesion. Fresh stimulated saliva was collected from the same healthy donor for each experiment. After washing in buffer solution, the slabs were transferred to culture plates containing 2 mL of ultrafiltered tryptone-yeast extract (LMW) broth with 1% sucrose and SM UA159 for initial bacterial adhesion. The inoculum was prepared from an exponential-growth culture of SM UA159 (100 μL of inoculum with the optical density of 1.6 was mixed with 50 mL of medium). After 8 h at 37°C, 10% CO_2_ the slabs were transferred to fresh LMW containing 1 mM glucose, where they were kept overnight (Fig 1). Over the next three days after the adhesion phase, biofilms were exposed 8×/day (8:00, 9:30, 11:00, 12:00, 13:30, 15:00, 16:00, and 17:30 h) to 10% sucrose solution for 3 min. Twice a day, after the first and last sucrose exposure, biofilms were treated with the assigned fluoridated solutions for 1 min and rinsed three times in 0.9% NaCl. LMW (supplemented with 1 mM glucose) was changed twice per day to fresh media, before the first and after the second fluoride treatment. The 8×/daily sucrose exposure and the night immersion in fresh medium induced demineralization periods of continuous pH drops followed by at night remineralization periods at neutral pH. The pH was measured to estimate biofilm acidogenicity at each change of medium as well as F and Ca concentrations. The baseline concentrations of F and Ca in the medium were also determined. After 96 h, biofilm formed on enamel and root dentine slabs were collected separately and analyzed for wet biofilm weight and soluble and bound-F concentration. Slabs surface hardness loss (%SHL) was evaluated as demineralization indicator. Fluoride uptake by enamel and root dentine was also determined as indicator of fluoride effect on the process of de- and remineralization.

### Biofilm harvesting and analysis

At 96 h of biofilm growth and in the morning of the last overnight incubation, slabs were removed from the culture medium, washed three times in 0.9% NaCl and the slabs of enamel and dentine were individually immersed in 1 mL 0.9% NaCl placed in pre-weighed microcentrifuge tubes. To detach biofilm from slabs, the tubes were sonicated for 30 s at 7 W (Branson, Sonifier 50, Danburry, Conn., USA) and slabs were removed to carry out the demineralization analysis [28]. Biofilm suspension was centrifuged at 10,000 *g*, 5 min at 4°C and the supernatant was collected for water soluble F analysis. The tube was again centrifuged and remnants of the supernatant was carefully vacuum-aspirated with a micropipette under a microscope to remove any solution. The biofilm pellet was weighed to obtain biomass (wet weight) and frozen for further analysis of bound F.

### Culture medium analyses

The medium collected twice/day (after the daily de- and remineralization periods) was analyzed for pH, and F and Ca concentration. Medium pH was determined using a microelectrode (Cole-Parmer Accumet, Vernon Hills, IL, USA) coupled to a pH meter (Procyon SA-720, Olímpia, SP, Brazil). For fluoride analysis, aliquots were buffered using TISAB II (1:1) and analyzed with an ion-selective electrode (Orion 96–09; Orion Research) and an ion analyzer (Orion EA-940; Orion Research), which had been previously calibrated with standard fluoride solutions prepared similarly to the samples. Ca was analyzed by colorimetric analysis using Arsenazo III [41], and the absorbance was read at 650 nm in 96-well microplates using a Multiskan Spectrum (Thermo Scientific) microplate reader.

### Determination of water soluble and bound F in biofilms

Soluble fluoride was measured in the supernatant of the saline extract. Aliquots were buffered using TISAB II (1:1) and analyzed with specific electrode as described above. Bound fluoride was extracted from the biofilm pellet by treatment with 0.5 M HCl (0.15 mL/10 mg bacterial wet weight) for 3 h [42]. The extract was centrifuged for 3 min at 16,000 *g* and F concentration in the supernatant was determined using a fluoride electrode adapted for microanalysis, after neutralization with 2.5 M NaOH and buffering with TISAB III [41]. For the analyses, the microelectrode was previously calibrated with F standards prepared as the samples.

### Demineralization determination

SH was used as an indicator of enamel [43] and dentine [25] demineralization. Three indentations, 100 μm apart, were made on the substrates before and after each experimental phase as described above in the slabs preparation section. Demineralization was expressed as percentage of surface hardness loss (%SHL) and calculated by the formula (baseline SH–SH after treatment)/baseline SH*100.

### Determination of F in enamel and dentine

The surfaces of enamel and root dentine slabs were isolated with wax, except the external surface where the indentations were made. The area exposed was determined and the slabs were immersed in 0.5 M HCl (3.57 mL/cm²) for 30 s under constant agitation (150 rpm) to remove an enamel or root dentine layer. The extract was buffered with an equal volume of TISAB II (pH 5.0), modified with 20 g NaOH/L [44]. The F concentration was determined with a specific F electrode as described for soluble F determination. Pi was measured in the acid extract [45] and the amount of enamel or dentine dissolved was calculated based on Pi concentration and density [44] of 17.4% and 2.92 g/cm^3^ for enamel and 13.5% and 2.14 g/cm^3^ for dentine. Fluoride concentration was expressed in μg F/g of enamel or dentine.

### Statistical Analysis

Data were analyzed by two-way ANOVA, considering the factors substrate (enamel or dentine) and F concentration (0, 150, 450, or 1,350 ppm F) using SAS system (SAS Institute Inc., version 9.2, Cary, NC, USA). Assumptions of homogeneity of variances and normal distribution of errors were checked for all response variables tested, and variables that did not satisfy these assumptions were transformed as suggested by the software. Regression analyses between %SHL and F concentration were also calculated. The significance level was set at 5%.

## Results

**Fig 2:**
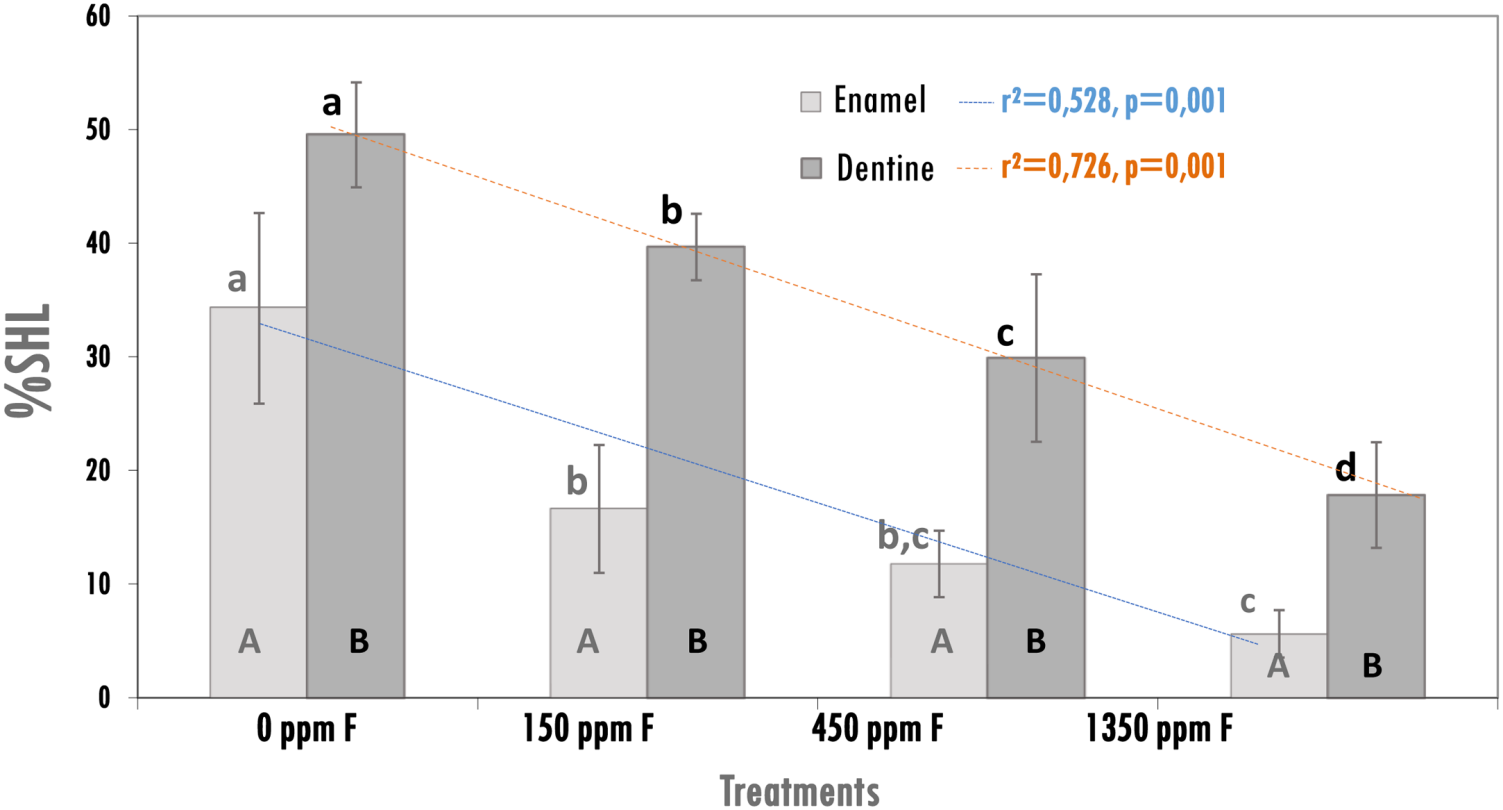
Percentage of surface hardness loss (%SHL) on enamel and dentine, according to the treatments (mean ± SD; n = 12), and values of regression analyses between treatments by substrate. Distinct capital letters indicate differences (p < 0.05) between enamel and dentine and distinct lower case letters indicate differences (p < 0.05) among fluoride treatments by substrate.

In terms of the effect of the treatments on enamel and dentine demineralization (Fig 2), a dose-response effect was seen for both dental substrates with a negative linear relationship between %SHL and fluoride concentrations of the treatments. Highest demineralization (p < 0.05) was found for the non-fluoride treatment group, either for enamel or dentine. In addition, demineralization was consistently higher in dentine than in enamel (p < 0.05) for all groups (Fig 2).

The data on fluoride concentration found in enamel and dentine (Table 1) were coherent with those observed for demineralization (Fig 2). A dose-response effect was observed for both dental substrates and higher fluoride concentration was found in dentine than enamel (p < 0.05)

**Table 1:** Fluoride concentration (μg F/g) on enamel and dentine, according to the treatments (mean ± SD; n = 12), and values of regression analyses between treatments by substrate.

Treatments^α^ (ppm F)	Fluoride concentration (μg F/g)	
	Enamel	Dentine
0 (control)	256.4±65.8 A,a	1105±331 B,a
150	439.4±200.0 A,b	3896±964 B,b
450	588.2±223.4 A,bc	4697±1113 B,bc
1,350	760.7±241.9 A,c	6133.±1280 B,c
*Linear regression*	r = 0.665; p < 0.001	r = 0.688p; p < 0.001

Distinct capital letters indicate differences (p < 0.05) between enamel and dentine (values within lines)

Distinct lower case letters indicate differences (p < 0.05) among fluoride treatments (values in columns for enamel and dentine

^α^ Concentrations to simulate brushing with toothpaste from low to high F concentration, considering the dilution (1:3) by saliva

All values transformed by log 10 to statistical analysis.

Thus, assuming the effect of fluoride on dentine was equivalent to that on enamel, dentine would have be treated with fluoride concentration 3× higher than enamel, 1,350 vs. 450 ppm F, as shown in Table 2.

**Table 2:** Reduction (%) of enamel and root dentine demineralization according to the treatments compared with their respective negative controls.

Treatment^α^ (ppm F)	*Reduction of demineralization (%)	
	Enamel vs control	Dentine vs control
0 (control)	-	-
150	46.8	21.3
450	60.9**	36.9
1,350	76.1	58.8**

* the calculation was relative to the respective control (%SHL with 0 ppm, 0% of reduction)

**Similar effect, about 60%, is observed with 450 ppm F for enamel and with 1,350 ppm F for dentine

^α^ Concentrations to simulate brushing with toothpaste from low to high F concentration, considering the dilution (1:3) by saliva.

**Fig 3:**
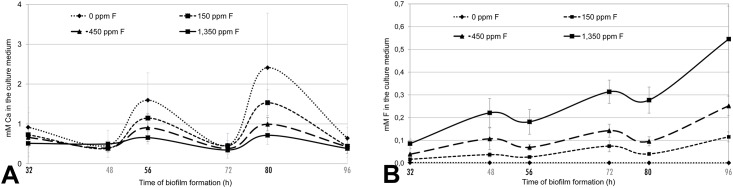
Calcium (A) and fluoride (B) concentration (mM) in the culture medium after cariogenic challenge period on the second day (32 h), third day (56 h), and on fourth day (80 h) and after the overnight period on the second day (48 h), third day (72 h), and on fourth day (96 h) (mean ± SD; n = 12).

The pH value of the culture medium, used as an indicator of biofilm acidogenicity, decreased after the daily sucrose exposure but differences among the treatments were not observed (S1 Fig). Also the wet weight of the grown biofilms was not different among treatments in each substrate (data not shown). F and Ca concentration in the culture medium (Fig 3) showed distinct patterns with fluoride concentration of the treatments, i.e., while fluoride increased, calcium decreased.

Regarding soluble fluoride concentration in biofilms, formed either on enamel or on dentine, the lowest and highest values (p < 0.05) were found for the negative control group and the treatment group with greater fluoride concentration, respectively (Table 3). However, for bound fluoride the difference among fluoride groups was not significant (p > 0.05) and only the biofilms formed on dentine differed from the non-fluoride group. Fluoride concentrations in biofilm formed on dentine by fluoride treatments were higher than those on enamel and statistically significant for the treatments with the highest fluoride concentration (Table 3).

**Table 3:** Soluble and bound fluoride in biofilms (μmol F/g) formed on enamel and dentine slabs, according to the treatments (mean ± SD; n = 12).

Treatments^α^ (ppm F)	Soluble F ^β^		Bound F	
	Enamel	Dentine	Enamel	Dentine
0 (control)	0.33±0.07A,a	^γ^0.30±0.09 A,a	0.17±0.08 A,a	0.12±0.07 B,a
150	1.93±0.49 A,b	2.25±0.39 A,b	0.22±0.09 A,a	0.31±0.31 A,b
450	2.89±0.54 A,c	4.37±1.03 B,c	0.23±0.07 A,a	0.32±0.15 A,b
1,350	4.65±1.07 A,d	7.63±1.53 B,d	0.28±0.11 A,a	0.46±0.30 B,b

Distinct capital letters indicate differences (p < 0.05) between enamel and dentine for soluble and bound fluoride (values within lines). Distinct lower case letters indicate differences (p < 0.05) among treatments (values in columns for each condition).

^α^ Concentrations to simulate brushing with toothpaste from low to high F concentration, considering the dilution (1:3) by saliva.

^β^ values transformed by log 10 to statistical analysis;

^γ^ outlier removed, value = 0.74 mol F/g biofilm.

## Discussion

This study was conducted because of the absence of studies on a validated cariogenic biofilm model to evaluate the dose-response effect of fluoride either on enamel or dentine demineralization, or simultaneously in both. The model was validated, and it enabled assessment of the anti-caries effect of fluoride on demineralization of dental substrates and also to analyze the effect of treatments on the biofilm. The dose-response effect of fluoride on reduction of demineralization was found for enamel and dentine (Fig 2). The concentrations of 150–1,350 ppm F used in the experiment simulates the dilution 1:3 that occurs in the oral cavity [38] when toothbrushing with toothpastes of low to high fluoride concentration. Therefore, this model could be used to estimate the anti-caries potential of innovative toothpaste formulations with low fluoride concentration [46] that could be recommended to control enamel caries in children, and formulations with high fluoride concentrations, that have been suggested to control root caries in elderly people [19, 21, 22]. Furthermore, the model could be used to estimate the anti-caries potential of fluoride on the cervical area in adults and elderly people, where dentine is exposed and enamel and dentine are at the same risk of caries.

Although a linear dose-response effect of fluoride concentration on reduction of demineralization has been found for enamel and dentine, the data clearly showed that the effect of fluoride was different on these dental substrates (Fig 2 and Table 2). Thus, while 450 ppm F produced a 60% reduction in demineralization of enamel, in dentine this effect at the same percentage could only be obtained with 1,350 ppm F. These findings can be explained by the fact that dentine is considered more susceptible to caries than enamel [7, 9] and moreover we found approximately twice more demineralization in dentine (49%) than in enamel (29%) not treated with fluoride, i.e., negative control group as shown in Fig 2. Our findings suggest that 3× more fluoride concentration would be necessary for dentine in order to achieve the same effect on percentage of reduction of demineralization as in enamel, which support clinical data suggesting that more fluoride would be required to control root caries [19, 21, 22] than that used for enamel. Also, the difference of fluoride effect on dentine compared with enamel explains why the combination of professional fluoride application with daily regular use of standard fluoride toothpaste would be more effective to control caries in dentine [25] but not in enamel [47, 48]. Contrary to enamel, on dentine the effect of the professional application in combination with daily fluoride toothpaste use was synergistic compared with the isolated effects of the two methods of fluoride application. The evidence is showing that fluoride does not have the same effect on dentine than enamel, then the recommendation should be that people with gingival recession brush their teeth with high fluoride concentration toothpaste.

The dose-response effect of fluoride concentration on reduction of enamel and dentine demineralization, evaluated by surface hardness (Fig 2) is also supported by the Ca concentration in the culture medium (Fig 3A). Ca is a chemical indicator of enamel and dentine demineralization and its concentration in the medium after the 8 sucrose exposures (demineralization period, times of 32, 56, and 80 h) was higher in the control group, where greater %SHL was found. Calcium concentrations were proportionally lower in the groups treated with fluoride. After the overnight remineralization period, when the biofilm stayed immerse in neutral medium for 48, 72, and 96 h, the difference between the groups decreased but the trend was maintained (Fig 3A). The results from fluoride concentration in the medium (Fig 3B) showed a pattern different from Ca and reflects the concentration of fluoride treatments. Given the neutral pH of the medium and the baseline concentration of calcium and fluoride, remineralization was allowed to occur during the overnight incubation period. Also, F released from the accumulated biofilm to the medium also contributed to the remineralization during overnight periods.

The data of soluble fluoride found in biofilm (Table 3) also give support to the dose-response effect of fluoride concentration on demineralization in either enamel or dentine (Fig 2). This concentration reflects the effect of the last treatment with fluoride because the biofilms were collected after the overnight remineralization period. This concentration also reflects the concentration found in the culture medium (Fig 3B). Concentrations of soluble fluoride in the biofilm was directly proportional to fluoride concentration of the treatments and help to explain the effect of fluoride on enhancement of remineralization.

We also found a dose-response effect between fluoride concentration of the treatments and fluoride concentration either in enamel or dentine (Table 1). This concentration of fluoride in enamel and dentine is a consequence of the mechanism of fluoride on the caries process. Fluoride interferes physicochemically with the caries process, reducing the demineralization of enamel and dentine when the pH falls after biofilm exposure to sugar and enhancing the remineralization process when pH rises again above the critical. Our model simulated the caries process because the biofilms were treated 8×/day with sucrose, when enamel and dentine were simultaneous subjected to demineralization, and during the night the slabs were subjected to remineralization because the biofilms were not treated with sucrose, and the pH was maintained around neutral values. Enamel and dentine were enriched with fluoride due to precipitation of fluoride apatite in enamel and dentine during the de-remineralizing process [49]. It is well known that dentine is more reactive to fluoride than enamel [20] but our findings show that dentine also gains more fluoride than enamel during the dynamics of the caries process (Fig 2). The present data extend those found by ten Cate et al. (1995), who showed that carious dentine incorporates more fluoride than enamel when treated with fluoride toothpaste and subjected to a chemical pH-cycling model. This higher amount of fluoride uptake (Table 1) in dentine during the caries process could be explained by two mechanisms: first, it would be a simple consequence by the fact that dentine is more demineralized than enamel and consequently more fluoride are changed with the minerals dissolved from dentine. Second, dentine has more amorphous calcium phosphate minerals than enamel and fluoride activates the phase transformation of these minerals in fluorapatite [9, 50].

The effect of fluoride found in the present study, reducing demineralization either in enamel or dentine, was essentially physicochemical, as described above. In fact, we did not find an effect of fluoride concentration on biofilm weight and acidogenicity, even when these are not at direct measured of microbial viability. Fluoride may have antimicrobial effects but it is concentration-dependent and fluoride concentrations either in biofilm treated with the greater concentration used (Table 3) or in the culture medium (Fig 3B) were below 10 ppm, the minimum fluoride concentration to inhibit enolase [51]. The findings gives support to the knowledge that the mechanism of action of fluoride on caries control is local and the antimicrobial effect of fluoride may be marginal when compared with the physicochemical effect [52, 53].

Regarding methodological aspects, the use of a single specie biofilm could be considered a limitation. However, in dental biofilm there are hundreds of bacterial species and it is impossible to simulate this complexity *in vitro*. Therefore, each model is developed for one specific reason and the present model was developed to evaluate the effect of fluoride on root and enamel caries. For this purpose, we improved a *S*. *mutans* biofilm model [32], which was validated to evaluate the effect of antibacterial substances on biofilm formation [28] and enamel demineralization [37], named cariogenic biofilm model [36] given the cariogenic properties of this bacterium. *S*. *mutans* is considered the most cariogenic bacteria in dental biofilm and it presents unique properties to metabolize sucrose, the most cariogenic dietary carbohydrate [54, 55]. However, this cariogenic biofilm model has not been validated in terms of dose-response to evaluate the effect of fluoride on either enamel or dentine, justifying the present publication. About the use of surface hardness as indicator of dentine mineral loss, it has been widely employed [25, 33, 34, 36, 39, 56–59] because there is a high correlation with transversal microradiography [25], which directly quantifies demineralization in dental enamel and also in dentine [60].

In summary, the findings consistently showed that this biofilm model is valid to evaluate the effect of fluoride in either enamel or dentine demineralization, or simultaneously in both. It should be emphasized that this model was validated in terms of dose-response effect of fluoride concentration to estimate the anti-caries potential of toothpaste formulations but it could also be useful in testing mouth rinse formulations. Furthermore, the findings support the clinical data, suggesting that higher fluoride concentrations are necessary to control caries of root dentine than of enamel.

## Supporting Information

S1 Fig pH of culture medium according to the time of biofilm formation and treatments. (TIF)
