# Supplementary figures and images for: Validation of a Cariogenic Biofilm Model to Evaluate the Effect of Fluoride on Enamel and Root Dentine Demineralization
Source: PLoS One. 2016 Jan 5;11(1):e0146478. doi: 10.1371/journal.pone.0146478 (PMC4712139; doi:10.1371/journal.pone.0146478)

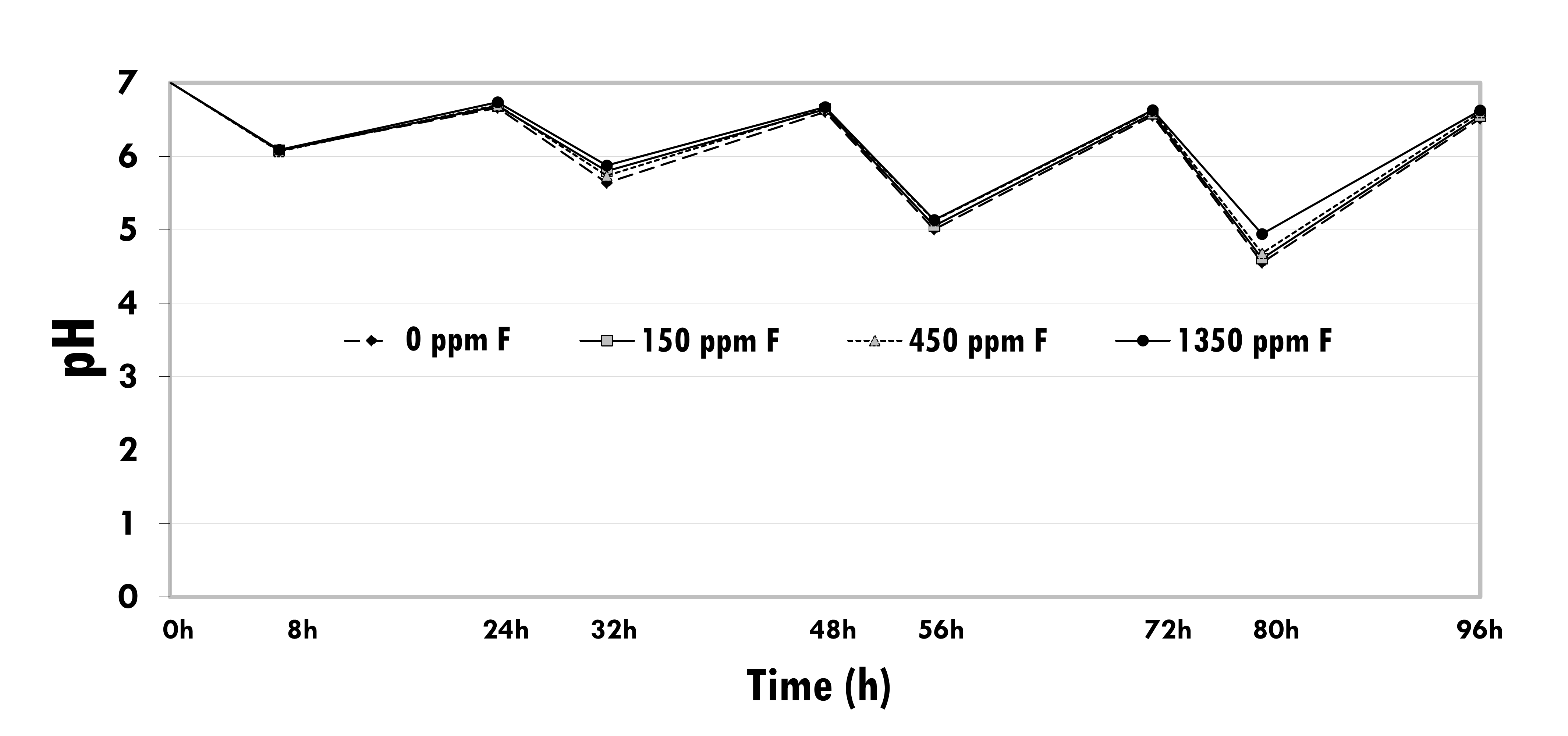

Supplement: S1 Fig — (TIF) [file pone.0146478.s001.tif]
